# Use of a pulmosphere model to evaluate drug antifibrotic responses in interstitial lung diseases

**DOI:** 10.1186/s12931-023-02404-7

**Published:** 2023-03-28

**Authors:** Kevin G. Dsouza, Ranu Surolia, Tejaswini Kulkarni, Fu Jun Li, Pooja Singh, Huaxiu Zeng, Crystal Stephens, Abhishek Kumar, Zheng Wang, Veena B. Antony

**Affiliations:** 1 Division of Pulmonary, Allergy, and Critical Care Medicine, Department of Medicine, University of Alabama at Birmingham, Birmingham, AL USA; 2 Superfund Research Center at The University of Alabama at Birmingham, 901 19Th St S, BMR2, Rm 404, Birmingham, AL 35294 USA; 3 Gainesville, Fl USA

## Abstract

**Background:**

Interstitial lung diseases (ILD) encompass a heterogenous group of diffuse parenchymal lung disorders characterized by variable degrees of inflammation and fibrosis. Pretherapeutic clinical testing models for such diseases can serve as a platform to test and develop effective therapeutic strategies. In this study, we developed patient derived 3D organoid model to recapitulate the disease process of ILDs. We characterized the inherent property of invasiveness in this model and tested for antifibrotic responses with an aim to develop a potential platform for personalized medicine in ILDs.

**Methods:**

In this prospective study, 23 patients with ILD were recruited and underwent lung biopsy. 3D organoid-based models (pulmospheres) were developed from the lung biopsy tissues. Pulmonary functioning testing and other relevant clinical parameters were collected at the time of enrollment and follow up visits. The patient derived pulmospheres were compared to normal control pulmospheres obtained from 9 explant lung donor samples. These pulmospheres were characterized by their invasive capabilities and responsiveness to the antifibrotic drugs, pirfenidone and nintedanib.

**Results:**

Invasiveness of the pulmospheres was measured by the zone of invasiveness percentage (ZOI%). The ILD pulmospheres (*n* = 23) had a higher ZOI% as compared to control pulmospheres (*n* = 9) (516.2 ± 115.6 versus 54.63 ± 19.6 respectively. ILD pulmospheres were responsive to pirfenidone in 12 of the 23 patients (52%) and responsive to nintedanib in all 23 patients (100%). Pirfenidone was noted to be selectively responsive in patients with connective tissue disease related ILD (CTD-ILD) at low doses. There was no correlation between the basal pulmosphere invasiveness, response to antifibrotics, and FVC change (Δ FVC).

**Conclusions:**

The 3D pulmosphere model demonstrates invasiveness which is unique to each individual subject and is greater in ILD pulmospheres as compared to controls. This property can be utilized to test responses to drugs such as antifibrotics. The 3D pulmosphere model could serve as a platform for the development of personalized approaches to therapeutics and drug development in ILDs and potentially other chronic lung diseases.

## Introduction

Interstitial lung diseases (ILD) encompass a group of parenchymal lung diseases that have similar clinical features and are characterized by impaired lung injury/repair response [1]. Idiopathic pulmonary fibrosis (IPF) is the commonest ILD, the exact cause of which remains unknown and is characterized by progressive lung function decline [2]. Similar, patterns of declining lung function are observed in other types of ILDs. ILDs have divergent physiological mechanisms, and are classified based on a broad array of etiologic phenotypes, such as autoimmune ILD, unclassifiable ILD, idiopathic nonspecific interstitial pneumonias and chronic hypersensitivity [3]. The common unifying pathologic manifestations of ILDs is the fibrosing nature of the disease, which overlaps with the pathogenesis of IPF. Anti-fibrotic therapies, Nintedanib and Pirfenidone show clinical benefits in patients with IPF [4, 5]. Based on the clinical and pathophysiological similarities with IPF, nintedanib was tested and approved as a therapy in patients with non-IPF progressive fibrotic ILD [6]. Studies suggest that pirfenidone may be beneficial in the treatment of unclassifiable progressive fibrosing ILDs and further investigation is warranted [7].

The development and progression of fibrosis is a prominent feature of IPF. We leveraged the invasive fibrotic nature of the disease to develop ex-vivo lung organoids ‘pulmospheres’ as a personalized and precision therapy model [8]. Similarly, underlying mechanisms and causes are disparate for non-IPF ILDs with the presence of progressive fibrosing phenotype. Current anti-fibrotic therapies, nintedanib and pirfenidone, are promising for the treatment of non-IPF ILDs [6, 7, 9, 10]. However, there is no certain way to compare and state the efficacy of available anti-fibrotic medications in an individual patient. We have utilized our pulmospheres for determining and comparing responses to nintedanib and pirfenidone in patients with non-IPF ILDs.

Pulmospheres are comprised of multi-type cells obtained from the lung tissue and majority of which are comprised of fibroblasts. The pulmospheres from patients with IPF have an increased number of myofibroblasts that pertains to the invasive phenotype in a fibrotic lung [8]. We used readouts for the invasiveness of the pulmospheres to compare the fibrotic phenotype and efficacy of anti-fibrotic drug treatment. In this current study, we have characterized the invasiveness of the pulmospheres of patients with non-IPF ILDs and tested the same pulmospheres for the comparisons and efficacy of anti-fibrotic effects of nintedanib and pirfenidone.

## Materials and methods

### Patient recruitment and clinical data

Lung tissue was obtained from subjects who were evaluated at the University of Alabama at Birmingham Interstitial Lung Disease Program. Lung tissue was obtained via surgical lung biopsies during the time of diagnosis in 17 patients or transbronchial biopsies in 5 patients. A total of 23 patients were enrolled in the study. The individual patient characteristics and diagnosis are enumerated in Table 1. The diagnosis of each individual patient was made by a multidisciplinary discussion based on the 2013 ATS/ERS guidelines [1]. Per usual clinical care, all subjects underwent pulmonary function tests that included FVC and DL_CO_ during their initial visit and subsequent follow up visit between 6 and 12 months.

**Table 1:** Pateint Data

Baseline characteristics	ILD N = 23	Donor explant controls N = 9	Time FVC% to biopsy (months)
Age (years ± SD)	60 ± 9.8	50 ± 7.8	
Sex (male, N %)	13 (57%)	4 (44%)	
Race			
White	18	7	
Black	5	2	
Type of interstitial lung disease (N, (%))			
Connective tissue disease	12 (52)		2.25 $\pm 1.75$
Unclassifiable	6 (26)		2.83 $\pm 1.20$
Smoking-related	4 (17)		1.55 $\pm 0.95$
Hypersensitivity pneumonitis	1 (4)		1.8

### Lung tissue and single-cell suspension preparation

Lung tissue samples from patients were washed with cold PBS solution. For approximately 6 mm × 6 mm tissue each, 5 ml of collagenase A was incubated with the tissue in a 50-ml tube for 30 min in a 37 °C water bath. The tube was gently agitated for a few seconds every 10 min during this incubation. PBS (20 ml) was added to the tube after 30 min of incubation. The tube was then vigorously shaken for 30 s to disassociate the lung tissue, and the resulting single-cell suspension was filtered through a 100-μm filter. The filtered single-cell suspension was verified by light microscopy and was centrifuged for 5 min at 300 g. The cell pellet was washed with complete DMEM (consisting of 10% FBS, penicillin, and streptomycin, and Glutamax, Invitrogen) and resuspended in 5 ml of ACK lysis buffer (catalog A1049201, Gibco) to lyse red blood cells. Tubes were incubated at room temperature for 5 min with occasional shaking. The reaction was stopped by diluting the ACK lysis buffer with 30 ml of PBS. Cells were centrifuged at 300 g at room temperature for 5 min. The supernatant was removed carefully, and the pellet was resuspended in complete growth media. Cells were plated in a T-25 flask. These passage 0 cells were used for the preparation of 3D spheroids.

#### Preparation of poly-HEMA–coated plates

Stock solution of poly-HEMA (120 mg/ml) was prepared in 95% ethanol. Working solution of poly-HEMA was obtained at final concentration of 5 mg/ml using 95% ethanol. The working solution was vortexed briefly for 30 s. A total 160-μl volume of working solution was added per well in 96-well U-bottom plates. Plates were left at room temperature in sterile laminar hood for 24 h (until the solution is evaporated). These coated plates were used for preparation of pulmospheres.

#### Preparation of pulmospheres

Primary lung cells were detached with trypsin and neutralized with 5 ml growth media. Cells were collected by centrifugation at 300*g* for 5 min at room temperature. The total cell pellet was resuspended in 2 ml complete DMEM medium. A portion of the cells was stained with trypan blue and counted to check for cell density and viability. 8000–10,000 cells (depending upon total number of cells and assays) were added per well in poly-HEMA–coated plate for both control and ILD pulmospheres. The plate was centrifuged (IEC-Centra 7R) at 200 g at room temperature for 1 min. Plates were incubated for 16 h at 37 °C in CO_2_ (5%) incubator. The resulting 3D spheroids were termed “pulmospheres.” We defined pulmospheres as in vitro 3D clusters of cells or spheroids derived exclusively from primary lung tissue and inclusive of lung cell types reflective of those in situ, in the patient.

#### Fluorescent automated cell sorting

Pulmospheres were disaggregated with trypsin (0.025%)/EDTA and washed with FACS buffer (5% FCS, 0.03% sodium azide–PBS).

#### Analysis of invasion by pulmospheres

Pulmospheres were formed following 16 h of incubation of seeded cells in poly-HEMA–coated plates. Analysis of invasion by pulmospheres was initiated immediately after the 16-h incubation. Collagen solution was prepared by mixing precooled (at 4 °C) 2.1 ml of collagen type I solution (3.98 mg/ml) (Corning) and 6.2 ml of DMEM (Invitrogen). NaOH (1 M) was used to set pH 7–7.4 of this collagen-DMEM solution. The solution was mixed gently by pipetting, avoiding the introduction of air bubbles. Of this solution, 50 μl/well was added to cover the bottom of flat-bottom 96-well plates. This bottom layer prevents contact of pulmosphere cells with the plastic of the well. We let the collagen set on a flat surface at 37 °C in a CO_2_ incubator for at least 2 h. Growth medium was removed from pulmospheres culture plate. To assess the responsiveness to antifibrotics, the ILD pulmospheres were tested for responsiveness with nintedanib and pirfenidone. Single pulmospheres were seeded onto the collagen-coated well with 100 μl of the collagen-DMEM solution with nintedanib (1 μM) or pirfenidone (100 μM) or media alone without test drug in a single well. These plates were incubated for 30 min at 37 °C in a CO_2_ incubator. 50 μl DMEM (with nintedanib [1 μM] or pirfenidone [1 mM] or without test drugs) was added to each well. Plates were incubated at 37 °C in a CO_2_ incubator overnight. In total, 5 pulmospheres were seeded without any test drug in collagen-DMEM solution into 5 individual wells; 5 pulmosphere were seeded with nintedanib-containing collagen-DMEM solution into 5 individual wells; and 5 pulmospheres were seeded with pirfenidone-containing collagen-DMEM solution into 5 individual wells for each patient tested. Images were acquired with a Carl Zeiss AxioCam color camera (Carl Zeiss Vision GmbH) and analyzed using AxioVision LE Imaging System software (Carl Zeiss Vision GmbH).

In the AxioVision LE Imaging System software, the area covered by the invading cells was selected and measured (H). The center mass of the pulmosphere was also outlined and measured (R). The percentage of total invaded area (ZOI%) was calculated by (H – R)/R × 100. Percentage invasion area was calculated. Measurement of fold change of ZOI was calculated as the ratio of ZOI% with treatment to ZOI% without treatment in vitro with antifibrotic drug.

#### Immunohistochemistry for Fibronectin-EDA

A part of VATs biopsies were fixed and embedded in paraffin. We de-paraffinized the lung sections and immunostaining them for Fibronectin-EDA as we described earlier [11]. We used fibronectin (at dilution 1:500) for the staining of the lung sections of the biopsies from the patients with ILDs. The intensities of DAB for the positive signal for fibronectin were calculated using NIH software Image J.

#### RT-PCR

Total RNA was obtained from control and ILD pulmospheres using RNeasy Mini Kit (Qiagen) and reverse transcribed using iScript Reverse Transcription SuperMix for RT- qPCR (Bio-Rad). Real-timePCR reactions were performed using SYBR Green PCR Master Mix (Life Technologies) and gene-specific primer pairs for human Co1a1 (forward-GATTCCCTGGACCTAAAGGTGC, reverse- AGCCTCTCCATCTTTGCCAGCA) and human GAPDH (forward-GTCTCCTCTGACTTCAACAGCG, reverse-ACCACCCTGTTGCTGTAGCCAA). Reactions were carried out for 40 cycles (95 °C for 15 s, 60 °C for 1 min) in a StepOnePlus Real Time PCR System (Life Technologies). Data was calculated by the ^ΔΔ^Ct method, normalized to β-actin, and expressed in AU.

#### Statistics

The results are presented as the mean ± SD unless specified otherwise. Comparisons between any two groups were performed using 2-tailed unpaired Student’s *t* tests and 2-tailed paired *t* tests. The comparisons within group were performed using paired *t* test. Response rate to each treatment was calculated. An exact 95% confidence interval around the response rate was calculated using the Clopper-Pearson method. Differences were considered statistically significant at *P* < 0.05.

## Results

We recruited 23 subjects who underwent a lung biopsy (surgical or transbronchial biopsy). 9 donor explants with no existing pulmonary disease were used to develop control pulmospheres. As previously described by our group, pulmospheres represent the in-vivo milieu of the lung microenvironment. They are 3D multicellular structures which consist of epithelial cells, macrophages, and fibroblasts embedded in extracellular matrix (ECM) proteins such as collagen type I, fibronectin-EDA, and collagen type IV. The baseline characteristics of the subjects are further described in Table 1.

### ILD lung pulmospheres are characterized by an invasive phenotype compared to controls

**Fig. 1:**
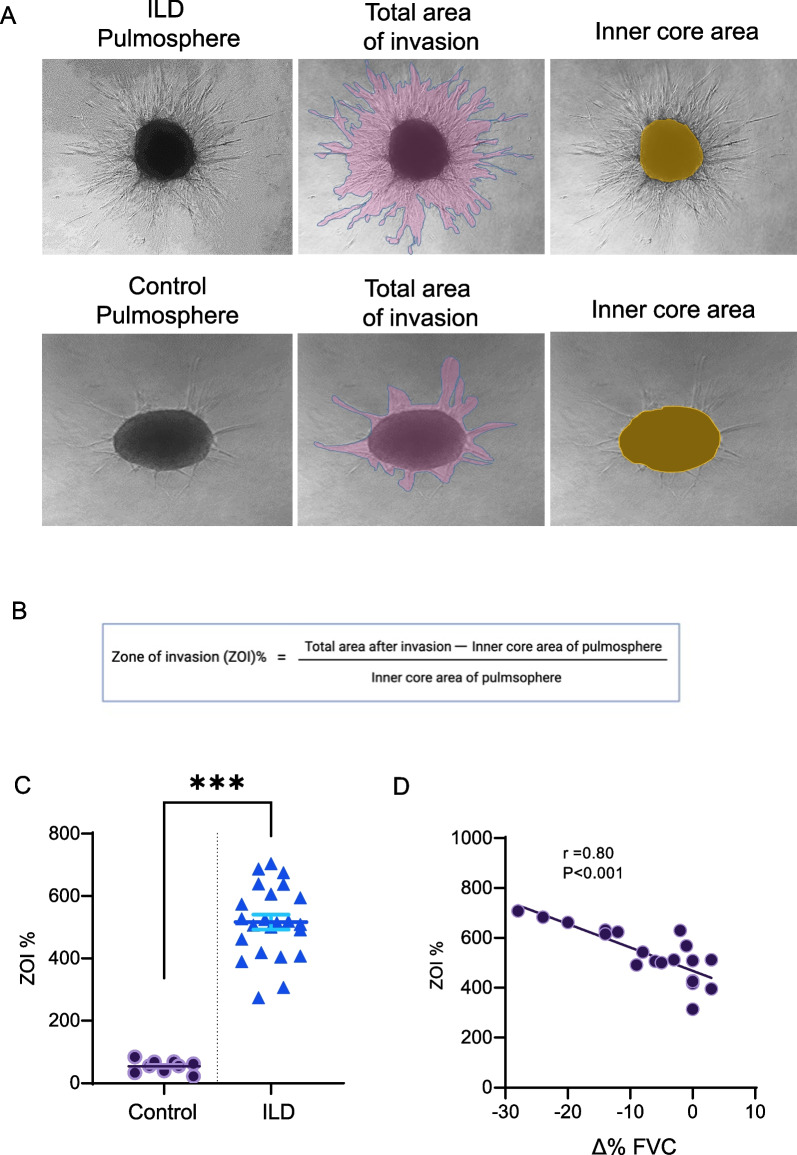
ILD lung pulmospheres are characterized by an invasive phenotype compared to controls. **A** Representative image of an invasive pulmosphere (ILD- top panel, control -bottom panel) depicting total area of invasion (purple) and inner core area (yellow). **B** Formula quantitating ZOI. **C** Scatter plot of control pulmospheres vs ILD pulmospheres depicting significantly higher invasiveness of the ILD pulmospheres n = 9 for control and 23 for ILD (Control Vs ILD, 54.63 ± 19.6 vs 516.2 ± 115.6, ***P value < 0.0001 estimated by Mann–Whitney test). **D** Simple linear regression analysis of ZOI% (invasiveness) with Δ %FVC (change in %FVC) showing positive correlation, P value < 0.001, R^2^ = 0.64, r = 0.80

Pulmospheres (ILD or control) were derived from primary lung cells by methods described above were evaluated for invasiveness by using the zone of invasiveness percentage (ZOI%), within each pulmosphere (Fig. 1A). The inner core area and the total area of each pulmosphere were measured and the zone of invasion (ZOI) was defined as the area covered by invasive cells. This was measured by subtracting the inner core area from the total area of each pulmosphere (Fig. 1B). The ZOI% of control (*n* = 9) and ILD (*n* = 23) pulmospheres were measured and revealed that the ILD pulmospheres had a higher ZOI% as compared to control pulmospheres 516.2 ± 115.6 versus 54.63 ± 19.6 respectively, P value < 0.0001 (Fig. 1C). Furthermore, we wanted to examine if the baseline pulmosphere invasiveness correlated with the decline in %FVC among patients with ILD. The %FVC values were obtained closest to the date of biopsy and on follow up between 6 to 12 months after the procedure. Simple linear regression revealed a positive correlation between change in %FVC (Δ %FVC) and pulmosphere invasiveness (r = 0.80, P-value < 0.001). This suggests that the inherent pulmosphere invasiveness may be a marker of progression in patients with ILD (Fig. 1D).

### Fibronectin and Collagen expression is increased in ILDs and correlates with pulmosphere invasiveness

**Fig. 2:**
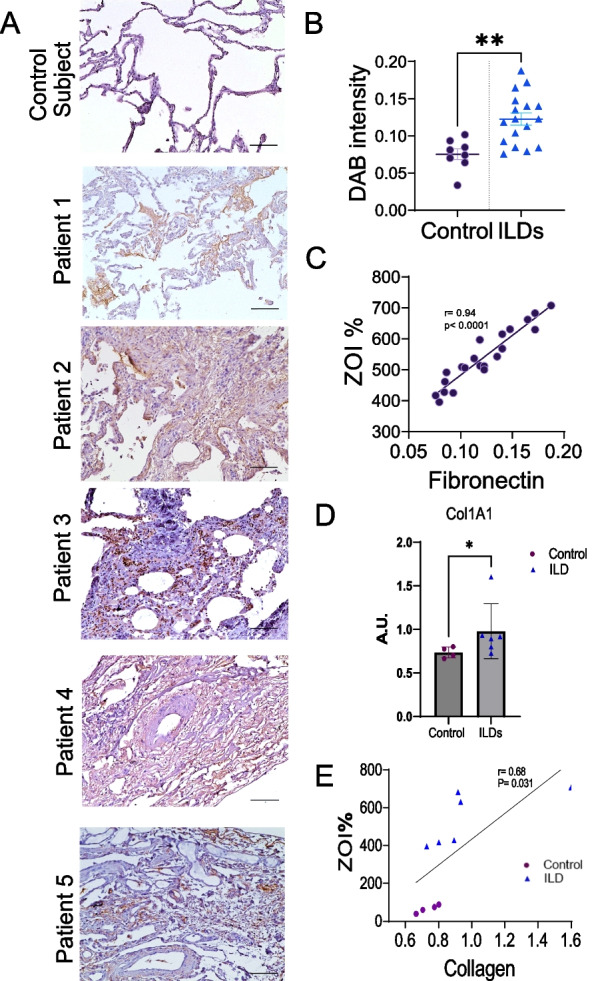
Fibronectin expression in increased in ILDs and correlates with pulmosphere invasiveness. **A** IHC staining showing expression of fibronectin in formalin-fixed, paraffin-embedded tissue section of a representative of 9 control subjects and 5 subjects with ILD. Scale bars: 100 μm. **B** Scatter plot of DAB fibronectin-EDA intensity control vs ILD tissue depicting significantly higher intensity in the ILD tissues. N = 9 for control subjects and 17 for ILD (Control Vs ILD, 0.075 ± 0.020 vs 0.122 ± 0.033, **P value = 0.0012 estimated by Mann–Whitney test). **C** Simple linear regression analysis of ZOI% (invasiveness) with fibronectin expression showing positive correlation, P value < 0.001, R^2^ = 0.88, r = 0.94. **D** RT-PCR showing col1A1 mRNA expression normalized to β-actin among Control Vs ILD pulmospheres,0.73 ± 0.06 vs 0.97 ± 0.31, P value = 0.0476 estimated by Mann–Whitney test. **E** Simple linear regression analysis of ZOI% (invasiveness) with collagen expression showing positive correlation, P value = 0.031, R^2^ = 0.45, r = 0.68

Fibronectin and Collagen are important ECM proteins which are expressed in non-IPF ILD’s [12–14]. Fibronectin levels are elevated in profibrotic monocytes, macrophages, and fibroblasts in scleroderma associated ILD [15, 16]. We analyzed VATS biopsy tissues of the control subjects and compared them to 5 ILD patients with IHC for fibronectin-EDA. There was increased expression of fibronectin in the ILD subjects compared to the control subject as measured by IHC for DAB fibronectin-EDA, Control Vs ILD, 54.63 ± 19.6 vs 516.2 ± 115.6, P value < 0.0001 (Fig. 2A, B). We further measured if the fibronectin expression noted in the patient biopsies correlated to the invasiveness of the individual pulmospheres. We found by simple linear regression there was a positive correlation between fibronectin expression and invasiveness (r = 0.94, P value < 0.0001), fibrosis suggesting that each pulmosphere can replicate the unique characteristics of each individual patient sample. Fibroblast invasion in lung pulmosphres has been known to enhance expression of collagen [17]. We performed RT-PCR to evaluate for Col1a1 mRNA expression among control and ILD pulmospheres. There was increased expression of Col1a1 mRNA in the ILD pumospheres compared to the control pulmosphres, Control Vs ILD,0.73 ± 0.06 vs 0.97 ± 0.31, P value = 0.0476 (Fig. 2D). There was positive correlation by simple linear regression between collagen expression and invasiveness (r = 0.96, P value = 0.031, Fig. 2E).

### Differential pulmosphere invasiveness in patients with ILD following exposure to nintedanib and pirfenidone

**Fig. 3:**
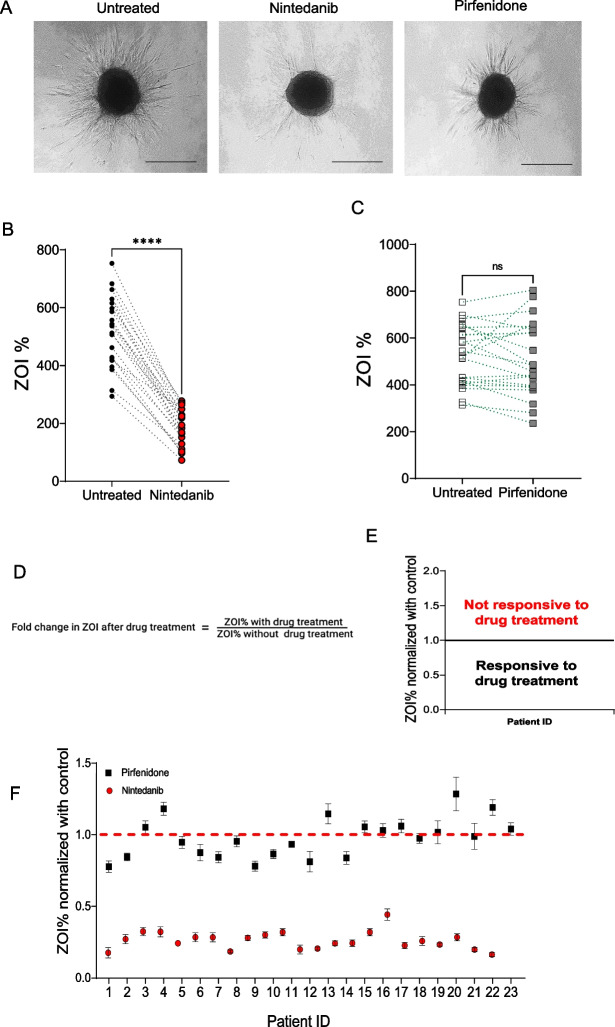

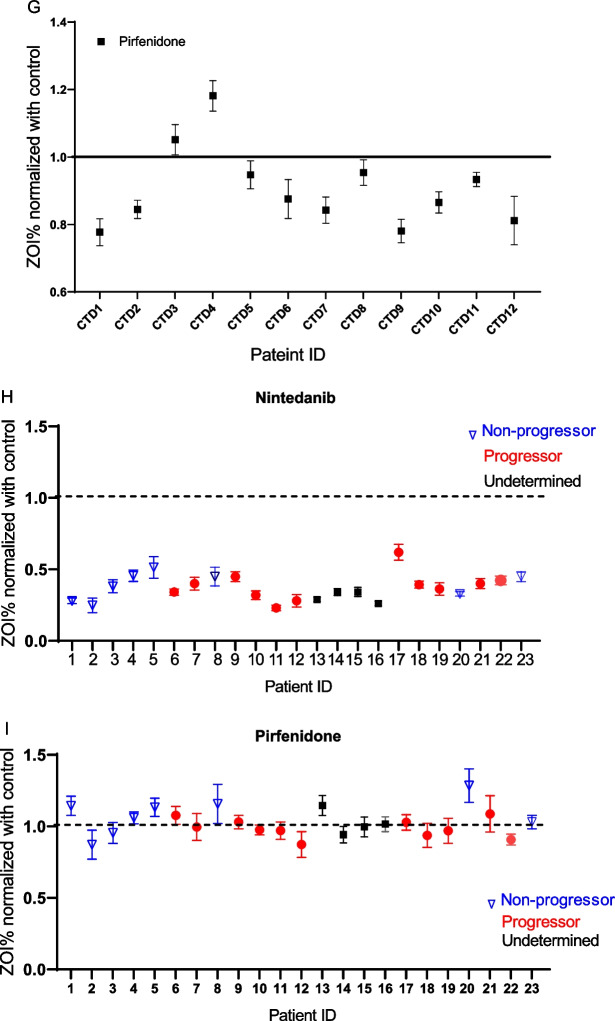
Differential pulmosphere invasiveness in patients with ILD following exposure to nintedanib and pirfenidone. **A** Representative image of an invasive pulmosphere compared to pulmosphere treated with nintedanib and pirfenidone. Scale bars: 100 μm. **B**, **C** Paired scatter plots depicting change in ZOI% of untreated (black) versus nintedanib (red) ILD pulmospheres, and untreated (white) versus pirfenidone (grey) treated ILD pulmospheres. Untreated (95% CI 465.1, 516.9 ± 119.9) vs nintedanib (95% CI 165.4,218.6, 192 ± 61.5, ****P value < 0.0001 by Wilcoxon test). Untreated (95% CI 471.4, 581.2 ± 126.9) vs pirfenidone (95% CI 444.5, 580.1, 512.3 ± 156.7, P value ns by Wilcoxon test). **D** Measurement of fold change of zone of invasion (ZOI) was calculated as the ratio of ZOI% with treatment to ZOI% without treatment in vitro with antifibrotic drug. **E** Median values of ZOI > 1 represent pulmospheres nonresponsive to the treatment, and median values of ZOI < 1 represent pulmospheres responsive to drug treatment. For each patient tested, 5 pulmospheres were seeded without either test drug; 5 pulmospheres were seeded with nintedanib (1 μM); and 5 pulmospheres were seeded with pirfenidone (100 μM). ZOI fold change values were obtained from the ratio of ZOI% with treatment to ZOI% without treatment of pulmospheres in vitro with antifibrotic drug for each patient. **F** Dot and whisker plot for ILD patient pulmospheres treated with nintedanib (red circles) and pirfenidone (black squares). Whiskers show maximum to minimum values. **G** Dot and whisker plot for CTD-ILD patient pulmospheres treated with pirfenidone (black squares). Dot and whisker plot depicting response to nintedanib (**H**) and pirfenidone (**I**) among ILD pulmospheres grouped into progressors (red circle), non-progressors (blue inverted triangle), and undetermined (no follow up data, black squares)

Antifibrotic drugs, nintedanib and pirfenidone, are FDA approved for the treatment of IPF [18]. Nintedanib and pirfenidone have been studied in patients with progressive fibrosing ILD which share clinicopathologic features with IPF [10, 19]. Decreased invasiveness of a pulmosphere treated by nintedanib and pirfenidone as compared to untreated pulmosphere from the same patient is illustrated in Fig. 3A. We measured the ZOI% of pulmospheres before and after treatment with nintedanib and pirfenidone. Nintedanib decreased invasiveness in pulmospheres measured by ZOI% (Untreated (95% CI 465.1, 516.9 ± 119.9) vs nintedanib (95% CI 165.4, 218.6, 192 ± 61.5, P value < 0.0001). The effect of pirfenidone on ZOI, although lower, was not statistically significant (Untreated (95% CI 471.4, 581.2 ± 126.9) vs pirfenidone (95% CI 444.5, 580.1, 512.3 ± 156.7, P value ns) (Fig. 3B, C).

The effects of these medications on the pulmospheres are represented as fold change of ZOI. This was calculated as the ratio of the ZOI% of the ILD pulmospheres with treatment to without treatment (Fig. 3D). Inhibition of invasiveness was defined as a ratio of less than one in our analysis. Conversely, the ratio of greater than one was defined as an increase in invasiveness (Fig. 3E). To account for inter-patient variability, we specified that a patient was responsive to treatment if the median value of fold change in ZOI was less than 1. We observed that ILD pulmospheres were responsive to pirfenidone in 12 of the 23 patients (52%). All 23 (100%) of the ILD pulmospheres were responsive to treatment with nintedanib (Fig. 3F). While further analyzing responses based on disease etiology, we found that 10 out of the 12 pulmospheres derived from CTD-ILD patients were response to treatment with pirfenidone (Fig. 3G). Next, we assessed if there were differences in anti-fibrotic reponses among progressors vs non- progressors based on FVC decline. Progressors were defined as individuals with an absolute FVC decline ≥ 5% within 12 months. Out of the 23 pateints, 4 patients were lost to follow up, 11 were progressors and 8 were non-progressors. Pulmospheres from both progressors and non-progressors were responsive to nintedanib (Fig. 3H). Pulmospheres from 6 out of the 11 progressors (54%), and 2 out of the 8 (25%) non-progressors were responsive to pirfenidone (Fig. 3I).Taken together, our results show the inhibitory effect of antifibrotic drugs on the invasiveness of pulmospheres and their utility as personalized models given the differential responses of each individual pulmosphere. Furthermore, nintedanib showed a robust response among progressors and non-progressors. Pirfenidone even at a low dose demonstrated a favorable response in 54% of progressors and was able to show a selective response in CTD-ILD derived pulmospheres.

### Correlation of basal invasiveness, antifibrotic response, and %FVC change

**Fig. 4:**
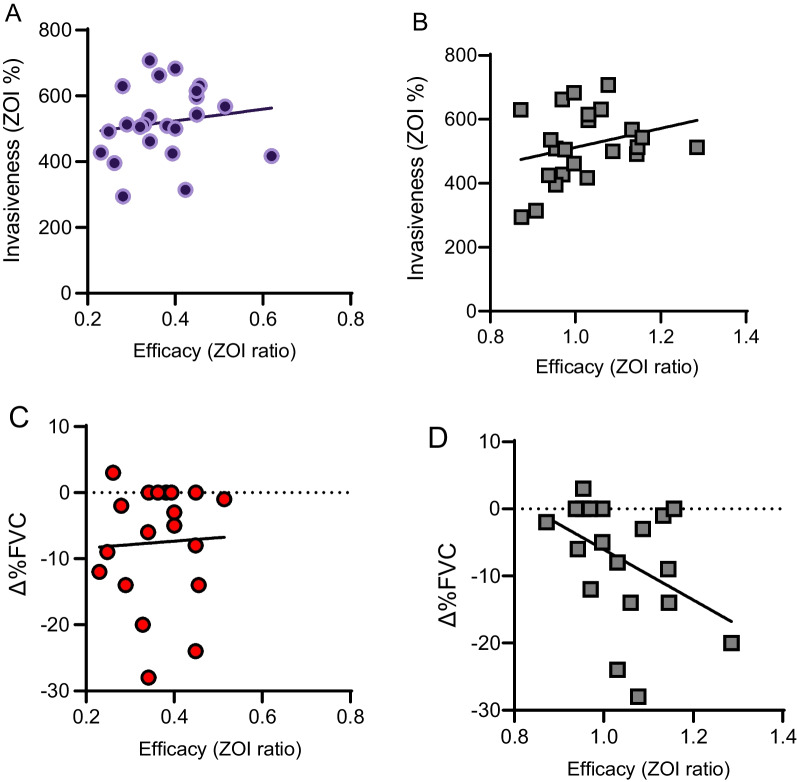
**A** Correlation analysis of ZOI% (invasiveness) with ZOI ratio(efficacy) with nintedanib treatment does not show correlation, P value = 0.49, R^2^ = 0.0224, r = 0.1496. **B** Correlation analysis of ZOI% (invasiveness) with ZOI ratio(efficacy) with pirfenidone treatment does not show correlation, P value = 0.204, R^2^ = 0.0755, r = 0.2747. **C** Correlation analysis of Δ %FVC (change in %FVC) with ZOI ratio(efficacy) with nintedanib treatment does not show correlation, P value = 0.444, R^2^ = 0.0329, r = 0.1813. **D** Correlation analysis of Δ %FVC (change in %FVC) with ZOI ratio (efficacy) with pirfenidone treatment does not show correlation, P value = 0.0850, R^2^ = 0.1558, r = 0.3947

Inherent invasiveness is a unique characteristic of each pulmosphere. This is measured by the ZOI% as described above. The ILD pulmospheres have demonstrated an invasive phenotype as compared to controls. We further investigated to ascertain if this invasive nature of the pulmosphere correlated with response to antifibrotic exposure. Invasiveness of the pulmosphere was measured by ZOI% ratio and antifibrotic response/efficacy to either pirfenidone or nintedanib was measured using ZOI ratio. There was no correlation of the basal pulmosphere invasiveness and response to nintedanib (r = 0.14, P value = 0.49), (Fig. 4A) and pirfenidone (r = 0.27, P value = 0.20) (Fig. 4B). Similarly, %FVC change (Δ %FVC) measured closest to the date of biopsy and on 6–12 month follow up (average, 7.8 ± 1.3 months) did not correlate with response to nintedanib (r = 0.18, P value = 0.44) (Fig. 4C) and pirfenidone (r = 0.39, P value = 0.0850) (Fig. 4D).

## Discussion

ILDs encompass heterogeneous disorders affecting the lung parenchyma with overlapping clinical, radiographic, and histopathologic manifestations. The heterogeneity of non-IPF ILDs necessitates the application of precision and personalized therapy. Recently, clinical trials have shown promising results for using nintedanib and pirfenidone as a therapeutic strategy for non-IPF fibrotic ILDs. Currently, nintedanib is FDA approved for the treatment of Progressive Pulmonary Fibrosis (PPF) [20, 21]. However, no tools or approaches are identified for the preference of the antifibrotic drug based on etiology, genetics, disease progression, or lifestyle. In this study, we demonstrate that the pulmospheres could serve as a precision and personalized medicine tool for patients with non-IPF ILDs. We utilized lung tissue obtained from the transbronchial biopsies and VATS to develop the pulmospheres and treated them with nintedanib and pirfenidone to test their differential effects on the invasiveness of the pulmospheres from an individual patient.

Pulmospheres are multi-cell type multicellular 3D structures, with the invasive properties recapitulating the in-vivo patient milieu. Previously, we utilized VATS lung biopsies for the preparation of single-cell suspension for the preparation of pulmospheres [8].The diagnosis of ILDs is based on the patient’s disease patient’s clinical history, HCRT, serological analysis, and at times confirmation by lung biopsy. In this study, we utilized VATS, and transbronchial biopsy samples to generate pulmospheres. We found that the transbronchial biopsies yielded enough tissue to develop pulmospheres. Transbronchial biopsies are less invasive,have lower risks compared to surgical lung biopsies and could be an alternative to surgical lung biopsies in certain ILDs [22]. Transbronchial lung cryobiopsy (TBLC) is another minimally invasive bronchospic technique which is increasingly used in the diagnosis of ILD’s [23]. Recent studies indicate that results acquired via TBLC are comparabale to surgical lung biopies [24]. Since the tissue sample obtained by TBLC is larger than traditional transbronchial biopsies, such samples would be amenable to developing pulmonspheres [25]. Our ability to generate pulmospheres from transbronchial biopsies; a less invasive alternative to the traditional surgical lung biopsy, confirms that pulmospheres are a pragmatic approach towards ‘precision medicine’ in the management of ILDs.

Pulmospheres are assimilated multi-type cellular structures with the functional ability of invasiveness. Invasiveness of fibroblasts is an inherent function for the homeostasis of tissue and wound repair; the dysregulation in this invasiveness of fibroblasts is recognized as a primary driver in the development of fibrotic diseases. Our pulmosphere model is based on the intemperate and aggressive invasiveness of fibroblasts of patients with lung fibrosis. As expected, our results indicated that pulmospheres from ILD patients display more invasiveness than controls. We found that the invasiveness of the pulmospheres was positively correlated with the expression of fibronectin in the tissue biopsies of the patients with ILDs. Collagen 1 levels were also increased in the ILD pulmospheres as compared to control pulmospheres. This is in line with our prior work wherein pulmospheres invasiveness enhances collagen expression [17]. We did not observe a differential result based on the sub-types of ILDs. However, we are limited by the small sample size in the study.

The property of invasiveness is unique to each patient endotype and correlated with the decline in FVC in the ensuing 6–12 months after obtaining the biopsy sample. Suggesting that the invasiveness of the pulmospheres, as measured by ZOI%, is unique to each patient and may correlate with clinical endpoints such as FVC decline. Patients with ILDs showed an increase in ZOI% with FVC decline as measured by Δ %FVC. It is to be noted that these differences follow up timings may affect absolute comparisons in disease progression secondary to our pragmatic approach in patient follow up and data collection. The increased invasiveness of fibroblasts and is an essential phenotype for severe fibrogenesis [26–30]. The ECM is a driver of progressive fibrosis [31]. Alterations in ECM proteins are noted in ILD’s such as hypersensitivity pneumonitis and scleroderma [32, 33]. The lung ECM interacts with multiple cell types and its dysregulation in chronic lung diseases including pulmonary fibrosis has been described [34]. Taken together, these ECM alterations and multicellular interactions are factors which influence fibroblast and overall pulmosphere invasiveness. ZOI% (invasiveness) of the pulmospheres positively correlated with the decline in lung function in ILD patients. Our presented work and prior studies in IPF establish that the pulmospheres may have predictive value for the prognosis of the diseases in patients with ILD.

Nintedanib and Pirfenidone differentially inhibited invasiveness of the pulmospheres in our study. We found decrease in the invasiveness of pulmospheres after treatment with nintedanib; however, the pulmospheres treated with pirfenidone showed only a moderate decrease in the invasiveness of the pulmospheres as compared to controls. The control group was the untreated pulmospheres of the same individual patient. Multiple factors such as treatment doses, heterogenous ILD population, and different mechanisms of drug action could have contributed to this result. We chose a lower dose of Pirfenidone based on pharmacokinetic data [35] compared to our prior study. Clinically, lower doses of pirfenidone in IPF have been shown to be as effective while minimizing side effects especially in the Asian population [36, 37]. Despite using a lower dose, we found that pirfenidone was effective in decreasing ZOI% compared to untreated controls among subjects with connective tissue disease related ILD. This result could mean that a lower dose of pirfenidone would be better tolerated by patients while possibly decreasing the rate of FVC decline in patients with connective tissue disease related ILD. Additionally, pulmosphere contain multiple cell types [8], and cell-cell communications as well as cell-autonomous and cell non-autonomous contributions, cytokines and ECM also affect the overall invasiveness of fibroblasts in the pulmosphere. The differential response to the pirfenidone could be explained by heterogenous responses to the cell types including alveolar epithelial cells and neutrophils [38–40].

An important application of our model is the ability to screen for therapeutic responses while minimizing the potential for adverse effects related to drug therapy, hence utilizing a more personalized approach. 3D organoid models recapitulate the in-vivo environment and can serve as physiologically relevant in-vitro models for pharmacological testing and research [17]. Among fibrotic ILDs, IPF has been extensively studied, and the role of many signaling pathways and cell types in the development of the disease is well known. Animal models and cell culture models do not accurately represent the complex microenvironment that occurs in the lung of patients with fibrotic lung disease [18, 19]. While several studies have been published using various organoid models in IPF, there is limited data on the use of organoid models as a predictive tool in assessing antifibrotic drugs [10]. Pulmonary organoid models have been explored in Hermansky-Pudlak syndrome, a genetic condition with a high prevalence of pulmonary fibrosis similar to IPF [20]. The development of alveolar organoids was performed by using patient derived iPSCs. Particularly, iPSCs were differentiated into NKX2-1+ lung epithelial progenitor cells through the 21-day differentiation following which the organoids were developed. Organoids represented altered genetic profile as expected in HPS patients, however these organoids were not purported to be a precision medicine tool. We believe that enrichment and genetic manipulations in the organoid forming cells deprives them of the exact representation of ongoing disease in the patients; however, they are better model systems for studying the specific mechanism in an individual patient for multifactorial disease. At this time, there is no data utilizing a heterogenous group of non-IPF fibrotic ILDs such as connective tissue disease-related ILD (CTD-ILD), smoking-related ILD, and hypersensitivity pneumonitis for the development of organoids or other precision medicine tools. Our model of pulmospheres for non-IPF ILDs encompasses the distinct and overlapping features of these heterogenous pulmonary diseases with a common underlying theme of progressive fibrosis. The approval of nintedanib; for the treatment of scleroderma associated ILD and PPF is proof of the mechanistic similarities among fibrotic ILDs [6, 21].

Antifibrotic therapies are associated with gastrointestinal, skin, and hepatic adverse events which can lead to discontinuation of therapy [41]. Personalized approaches to therapy can avoid patient exposure to adverse effects and/or ineffective therapies. For example, the patient numbers 1,2,5,6,7,8,9,19,11,12,14 and18 demonstrated that nintedanib and pirfenidone, both were effectively inhibiting the invasiveness of their pulmospheres (Figure. 3F). Our future studies will explore correlation of clinical and pulmosphere responses to antifibrotics in ILD’s. If successful, these models can be used to select potential therapeutic options. Hence, our 3D-organoid model ‘pulmosphere’ can serve as a platform for future therapeutics development and testing in this area of unmet clinical need.

Our study was conducted prior to the FDA approval of nintedanib for progressive fibrosing ILD’s and hence none of the subjects were on antifibrotic medications during the follow up period. This further validates the correlation between the FVC decline and pulmosphere invasiveness in patients with fibrotic ILDs since there was no further *in vivo* antifibrotic effect that may have affected the results.

Our current work builds upon of our experience with developing the pulmosphere model in in IPF [42]. We are limited by our sample size, and inability to utilize TBLC samples which is has become a more widely accepted method of obtaining tissue samples in ILDs. Moreover, clinical correlation between lung function on antifibrotic therapy and in-vitro response were not explored. Additionally, some patients were started on immunosuppressive therapy after their diagnosis, a potential confounder.

Overall, the clinical relevance of our study is important in view of the paradigm shift that has occurred in the treatment of PPF with the approval of nintedanib for this patient phenotype. Newer drug development and expanding the domain of current therapies are the need of the hour; especially for patients that show truncated response towards current antifibrotic therapy. Preclinical and predictive testing of novel therapies has the potential to deliver a more personalized approach to patients that are unresponsive to nintedanib, and pirfenidone, and the patients who show adverse effects to both the drugs. We performed these tests before the clinical trial studies showing the efficacy of antifibrotics in the treatment for non-IPF ILDs. We also developed pulmospheres from transbronchial lung biopsies recognizing that not all patients with ILD would routinely need a surgical lung biopsy as part of clinical care. Our results validate the potential use of our ‘precision therapy’ model for repurposing anti-invasion/ proliferation drugs and testing novel anti-fibrotic drug molecules for the treatment of ILDs. It can also help further understand the pathobiology of ILDs making it an ideal platform for future research and drug development.

## Data Availability

All data generated or analyzed during the study are included in this article.

## Abbreviations

ILD: Interstitial lung disease
IPF: Idiopathic pulmonary fibrosis
FVC: Forced vital capacity
DL_CO_: Diffusing capacity of lungs for carbon monoxide
ZOI%: Zone of invasiveness percentage
Δ% FVC: Change in % FVC
ECM: Extracellular matrix
PF-ILD: Progressive fibrosing ILD
PPF: Progressive Pulmonary Fibrosis
ZOI: Zone of invasiveness
